# Supplementary material for: Disulfiram/Copper Induces Immunogenic Cell Death and Enhances CD47 Blockade in Hepatocellular Carcinoma
Source: Cancers (Basel). 2022 Sep 28;14(19):4715. doi: 10.3390/cancers14194715 (PMC9564202; doi:10.3390/cancers14194715)
Supplement: Supplementary file 1 [file cancers-14-04715-s001.zip › cancers-1934320-supplementary.pdf]

Figure S1

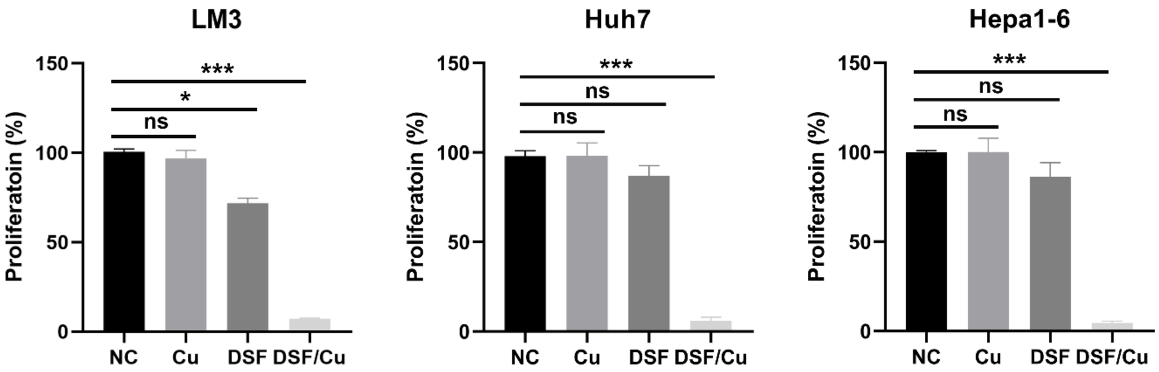

Figure S1. Cell viability of LM3, Huh7 and Hepa1-6 cells treated with DSF (0.2μM)/Cu (1μM) for 24h.

Figure S2

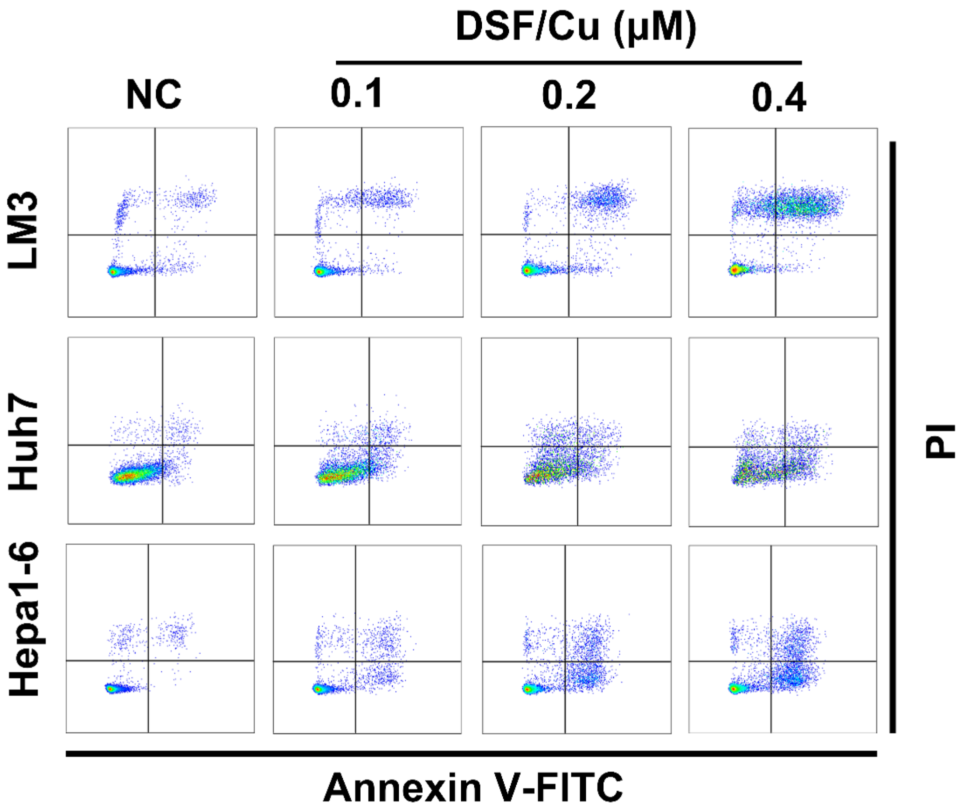

Figure S2. Apoptosis analysis of LM3, Huh7 and Hepa1-6 HCC cells treated with different concentration of DSF/Cu.

Figure S3

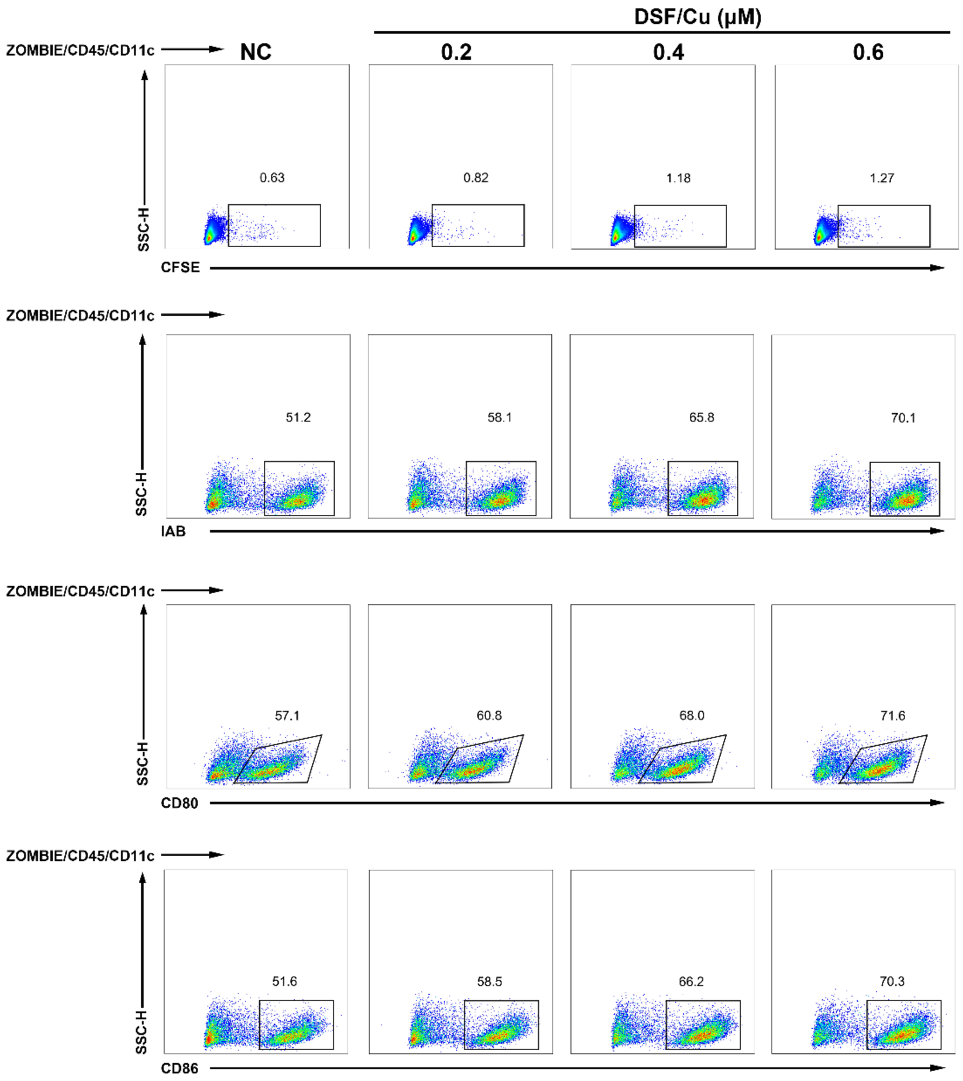

Figure S3. The expression of CFSE, I-Ab, CD80 and CD86 of CD11c+ DC cells after coculture were detected by flow cytometry.

Figure S4

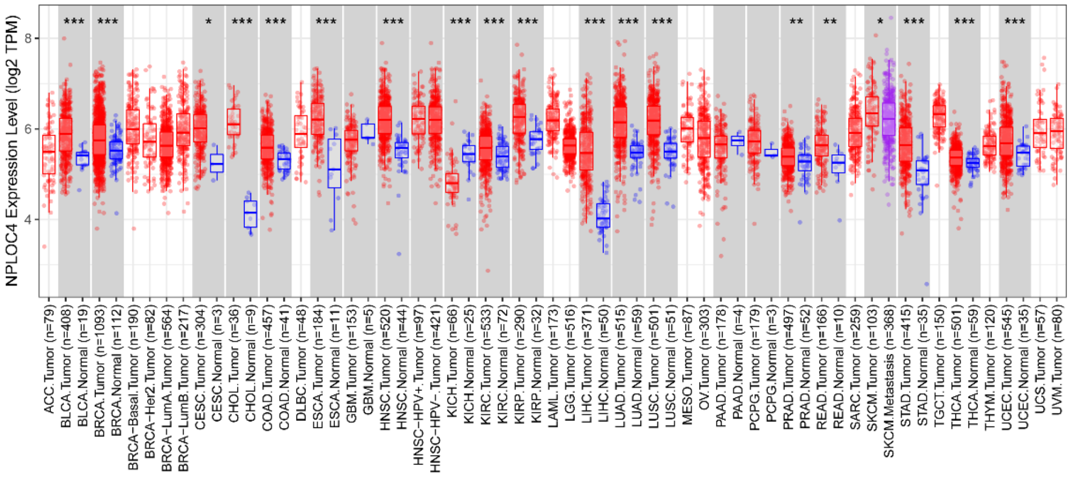

Figure S4. In the GEPIA database, the expression of NPL4 in tumor tissues compared with normal tissues.

Figure S5

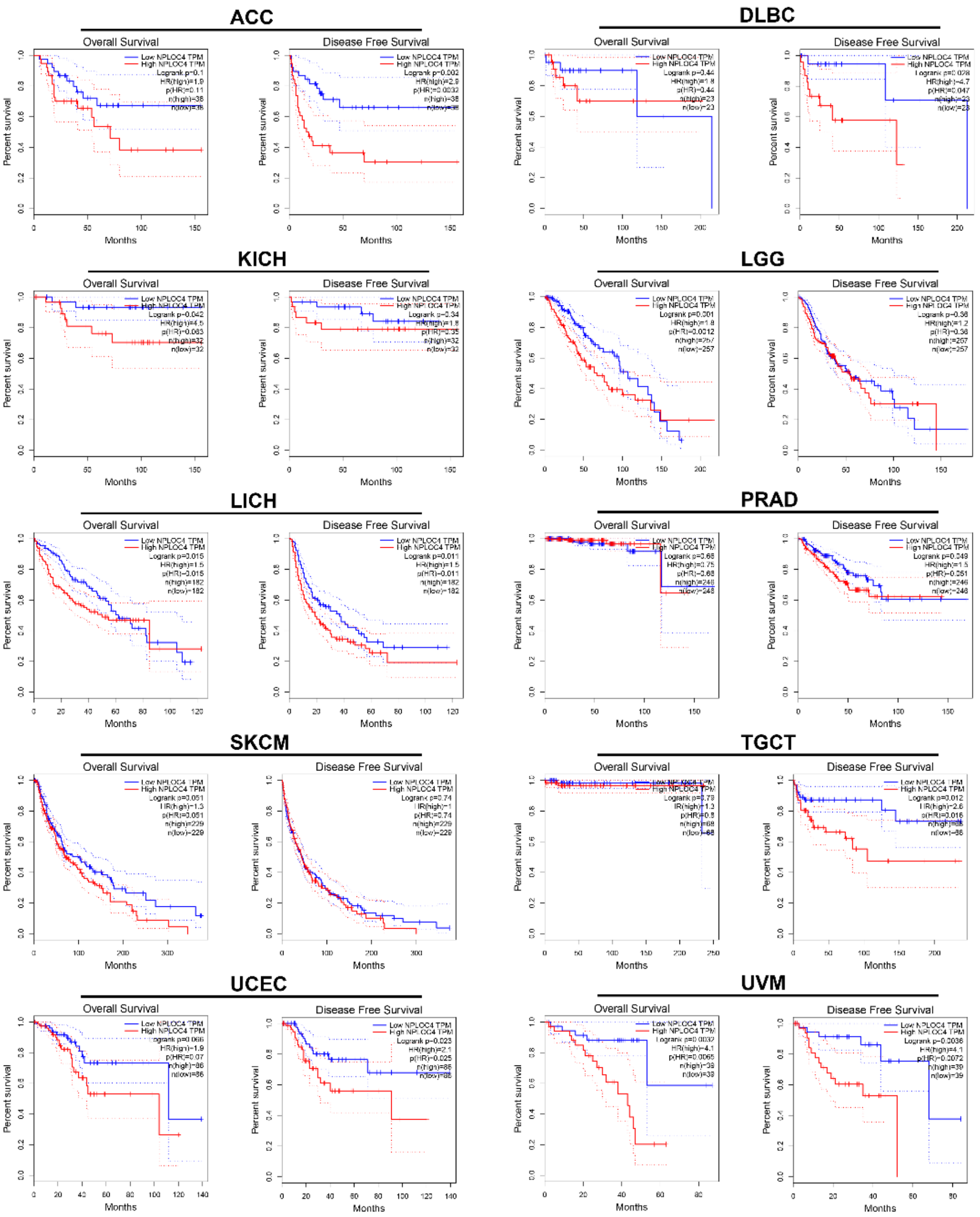

Figure S5. Kaplan–Meier survival curves comparing high and low expression of NPL4 in different cancer types in GEPIA.

Supplementary methods

Cell culture

HCC-LM3, Huh7 and Hepa1-6 cells were obtained from America Type Culture Collection (Manassas, VA) in 2017. Huh7 were cultured in Modified Eagle Medium (MEM, Life Technologies, Gaithersburg, MD) supplemented with 10% FBS, 1% penicillin/streptomycin solution. HCC-LM3 and Hepa1-6 cells were cultured in Dulbecco's Modified Eagle Medium (DMEM, Life Technologies, Gaithersburg, MD supplemented with 10% FBS, 1% penicillin/streptomycin solution. The Cell line authentication was conducted at the same time.

Cell viability assay

The concentration of CuGlu was 1uM in this study. Cell viability was detected with CCK8 kit according to the manufacture's instruction. Cells were seeded on 96-well plates and treated with designed treatments, and then 10 μL CCK8 reagent was added to each well (containing 100 μL of medium), for further incubation at 37 °C for 1 h. Finally, the absorbance at 450 nm was measured by microplate reader. The cell viability in each group was calculated as the ratio to vehicle control. The EdU assay kit (UE, China) was used to determine the proliferation rate of cells according to the manufacturer's instructions.

Colony formation assays

Cells (2×10^3) were plated in plates for 7 to 10 days and treated with the designed concentration of DSF/Cu for another 3 days. The colonies were fixed with 4% paraformaldehyde and stained with 0.1% crystal violet for 30 min. The colony numbers were counted with Image J software.

Immunoblotting

Proteins were extracted (Minute SN-001, Invent Biotechnologies, Inc, Beijing, China) and subjected to 8%-15% SDS-PAGE, and transferred to PVDF membranes, followed by blocking with 5% milk-TBST and incubated with primary antibodies overnight. HRP-conjugated anti-mouse or anti-rabbit antibodies were used as the secondary antibodies, and the antigen-antibody reaction was visualized by enhanced chemiluminescence assay (ECL, Thermo, Waltham, MA).

Immunofluorescence

Cells were seeded on plastic inserts in 12-well dishes. Next day cells were treated with compounds at indicated concentrations and subsequently pre-extracted (0.1% Triton X 100 in PBS, for 2 min) and fixed with 4% formaldehyde for 15 min at room temperature, washed with PBS and permeabilized with 0.5% Triton X-100 in PBS for 5 min. After PBS washes, the cells on the plastic inserts were then immunostained with anti-NPL4 (sc-365796, santa cruz) overnight in 4 °C, followed by a PBS wash and staining with fluorescently-conjugated secondary antibody for 60 min at room temperature. Nuclei were visualized by DAPI staining at room temperature for 2 min

Flow Cytometry Staining

Mice were sacrificed at the indicated times, and tumor lesions were harvested and minced to prepare single-cell suspensions using the Tumor Dissociation Kit (Miltenyi Biotec, 130-096-730). Mononuclear cells were isolated from the single cell suspension by OptiPrep Density Gradient Medium (Sigma-Aldrich, St. Louis, MO). The cells were stained with antibodies. All the reagents were from eBioscience (San Diego, CA) unless otherwise indicated.

siRNA

NPL4 siRNA and siRNA NC were designed by RiboBio (Guangzhou, China). NPC cells were seeded into 6-well plates at 2.5×10^5 cells/well and incubated overnight to reach 60–70% confluence for transfection. Transfections were performed using Lipofectamine 3000 (Invitrogen, Carlsbad, CA, USA) according to the manufacturer’s protocol. The target sequences used in the siRNA were as follows

Gene	5’-3’
NPL4 siRNA-1	GGTACCAGGTGTCCAATCA
NPL4 siRNA-2	GACCTCGTCTCAGAAGATA

Dendritic cell activation and phagocytosis assays

Bone marrow-derived dendritic cells (BMDCs) extracted from the thigh-bone of C57BL/6 mice were cultured in RPMI 1640 medium supplemented with 10% FBS, 1% penicillin/streptomycin solution along with GM-CSF (25 ng/ml, 315-03, PeproTech) for 7 days for further experiment. Hepa1-6 cells were treated with DSF/Cu for 24 h. For phagocytosis assays, treated hepa1-6 cells were labeled with 5 μM CFSE (Biolegend) and cultured with DCs at a 1:1 ratio for 4 h. For DC activation assays, treated hepa1-6 cells were cultured with DCs at a 1:1 ratio for 24 h.
