# Supplementary figures and images for: Disulfiram/Copper Induces Immunogenic Cell Death and Enhances CD47 Blockade in Hepatocellular Carcinoma
Source: Cancers (Basel). 2022 Sep 28;14(19):4715. doi: 10.3390/cancers14194715 (PMC9564202; doi:10.3390/cancers14194715)

**Figure 3C**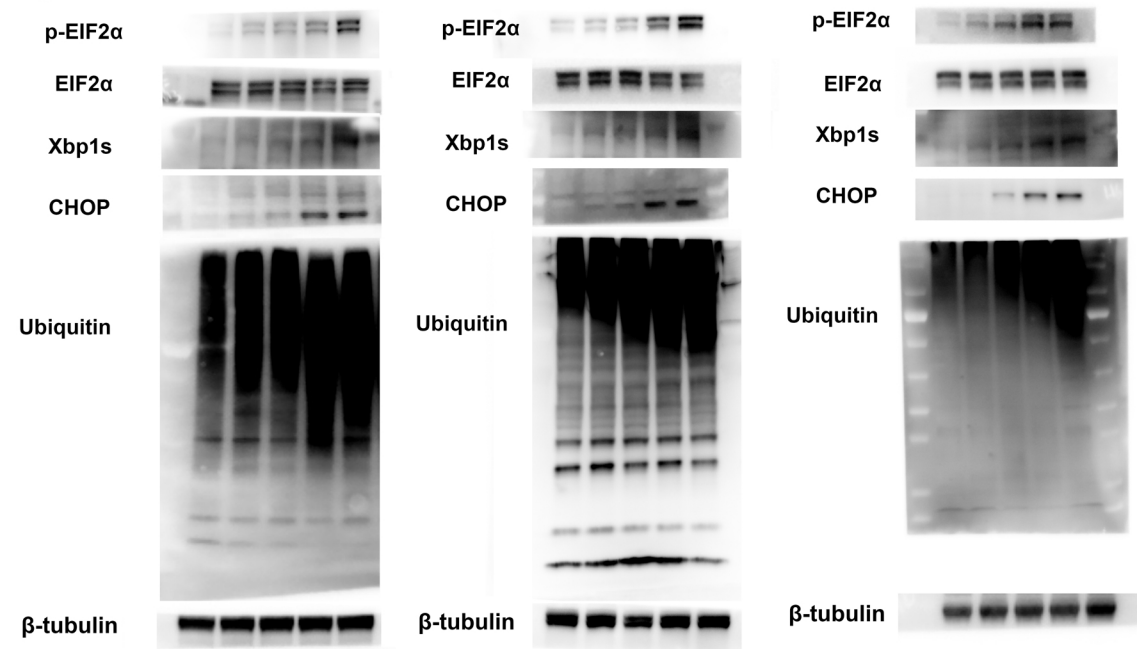**Figure 3E**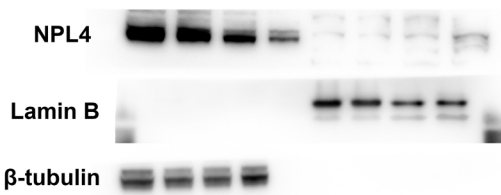**Figure 4A**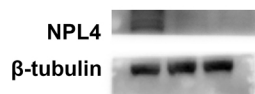**Figure 4I**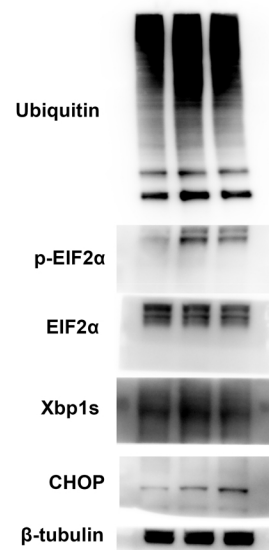

Supplement: Supplementary file 1 [file cancers-14-04715-s001.zip › Figure S6.pdf]
